# Experimental Combination Therapy with Amiodarone and Low-Dose Benznidazole in a Mouse Model of Trypanosoma cruzi Acute Infection

**DOI:** 10.1128/spectrum.01852-21

**Published:** 2022-02-09

**Authors:** Juliana Magalhães Chaves Barbosa, Yasmin Pedra Rezende, Tatiana Galvão de Melo, Gabriel de Oliveira, Cynthia Machado Cascabulho, Evelyn Nunes Goulart da Silva Pereira, Anissa Daliry, Kelly Salomão Salem

**Affiliations:** a Laboratório de Biologia Celular, Instituto Oswaldo Cruz, Rio de Janeiro, Brazil; b Laboratório de Ultraestrutura Celular, Instituto Oswaldo Cruz, Rio de Janeiro, Brazil; c Laboratório de Inovações em Terapias, Ensino e Bioprodutos, Instituto Oswaldo Cruz, Rio de Janeiro, Brazil; d Laboratório de Investigação Cardiovascular, Instituto Oswaldo Cruz, Rio de Janeiro, Brazil; Weill Cornell Medicine

**Keywords:** *Trypanosoma cruzi*, Chagas disease, amiodarone, benznidazole, cardiac function

## Abstract

Chagas disease (CD), caused by Trypanosoma cruzi, affects approximately 6 to 7 million people in Latin America, with cardiomyopathy being the clinical manifestation most commonly associated with patient death during the acute phase. The etiological treatment of CD is restricted to benznidazole (Bz) and nifurtimox (Nif), which involve long periods of administration, frequent side effects, and low efficacy in the chronic phase. Thus, combined therapies emerge as an important tool in the treatment of CD, allowing the reduction of Bz dose and treatment duration. In this sense, amiodarone (AMD), the most efficient antiarrhythmic drug currently available and prescribed to CD patients, is a potential candidate for combined treatment due to its known trypanocidal activity. However, the efficacy of AMD during the acute phase of CD and its interaction with Bz or Nif are still unknown. In the present study, using a well-established murine model of the acute phase of CD, we observed that the Bz/AMD combination was more effective in reducing the peak parasitemia than both monotherapy treatments. Additionally, the Bz/AMD combination reduced (i) interleukin-6 (IL-6) levels in cardiac tissue, (ii) P-wave duration, and (iii) frequency of arrhythmia in infected animals and (iv) restored gap junction integrity in cardiac tissue. Therefore, our study validates AMD as a promising candidate for combined therapy with Bz, reinforcing the strategy of combined therapy for CD.

**IMPORTANCE** Chagas disease affects approximately 6 to 7 million people worldwide, with cardiomyopathy being the clinical manifestation that most commonly leads to patient death. The etiological treatment of Chagas disease is limited to drugs (benznidazole and nifurtimox) with relatively high toxicity and therapeutic failures. In this sense, amiodarone, the most effective currently available antiarrhythmic drug prescribed to patients with Chagas disease, is a potential candidate for combined treatment due to its known trypanocidal effect. In the present study, we show that combined treatment with benznidazole and amiodarone improves the trypanocidal effect and reduces cardiac damage in acutely T. cruzi-infected mice.

## INTRODUCTION

Chagas disease (CD) is caused by the flagellate protozoan Trypanosoma cruzi, described in 1909 by the Brazilian physician Carlos Chagas (1). CD is listed by the World Health Organization (WHO) as one of the 20 neglected tropical diseases and affects approximately 6 to 7 million people worldwide, primarily in Latin America (2, 3). The clinical manifestation most frequently associated with death in acute patients is chagasic cardiomyopathy (CC) (4). The most common clinical findings in patients with CC are (i) electrocardiographic (ECG) abnormalities, (ii) lymphoid inflammatory infiltration, and (iii) myocyte destruction and, consequently, reparative fibrosis in cardiac tissue (5).

The etiological treatment of CD is still limited to two nitroheterocycles, benznidazole (Bz) and nifurtimox (Nif) (6). Their efficacy varies with (i) the phase of infection, (ii) the dose and duration of treatment, (iii) the immune status, and (iv) the geographic origin of the patient, as some T. cruzi strains are naturally resistant to nitroderivatives (7, 8). Furthermore, the severe side effects of Bz and Nif and their limited efficacy in the chronic phase justify the urgent need for treatment strategies for CD management (9). With the aim of improving etiological treatment, repurposing and combined therapy for CD are being intensively studied (10–12). The combined use of drugs allows attacking different targets in the parasite, improving host defense, and avoiding drug resistance, thus enhancing efficacy (13).

Amiodarone (AMD), a class III antiarrhythmic agent, is the most frequently used drug for the treatment of cardiac arrhythmias in CD patients (14). The trypanocidal activity of AMD has been demonstrated both *in vitro* and *in vivo* (15). The *in vitro* cardioprotective effect of AMD has been reported using T. cruzi-infected cardiomyocytes, and reduced proliferation of intracellular amastigotes, recovery of the spontaneous contractility of cardiac cells, F-actin fibrillar organization and connexin-43 distribution have been observed (16). In the BENEFIT study, patients with chronic chagasic cardiomyopathy (CCC) treated with Bz showed a reduction in the parasite burden, but cardiac disease progression was not prevented; however, interestingly, those treated concomitantly with AMD and Bz presented a reduction in both hospitalization incidence and death risk due to cardiovascular complications (17, 18).

Therefore, in the present study, we tested the hypothesis that combined treatment with Bz/AMD might be more effective in eliminating the parasite and reducing cardiac damage than each individual drug. To this end, we used a well-established mouse model of acute T. cruzi infection that reproduces the pathological features of human disease (19, 20) and evaluated the effect of Bz/AMD and each drug on parasitemia, cardiac inflammation and fibrosis, ECG abnormalities, and gap junction integrity. We propose the combination of Bz/AMD as an alternative treatment for acute CD.

## RESULTS

### Administration of Bz/AMD reduces the parasite load and increases the survival rate of T. cruzi-infected mice.

**FIG 1:**
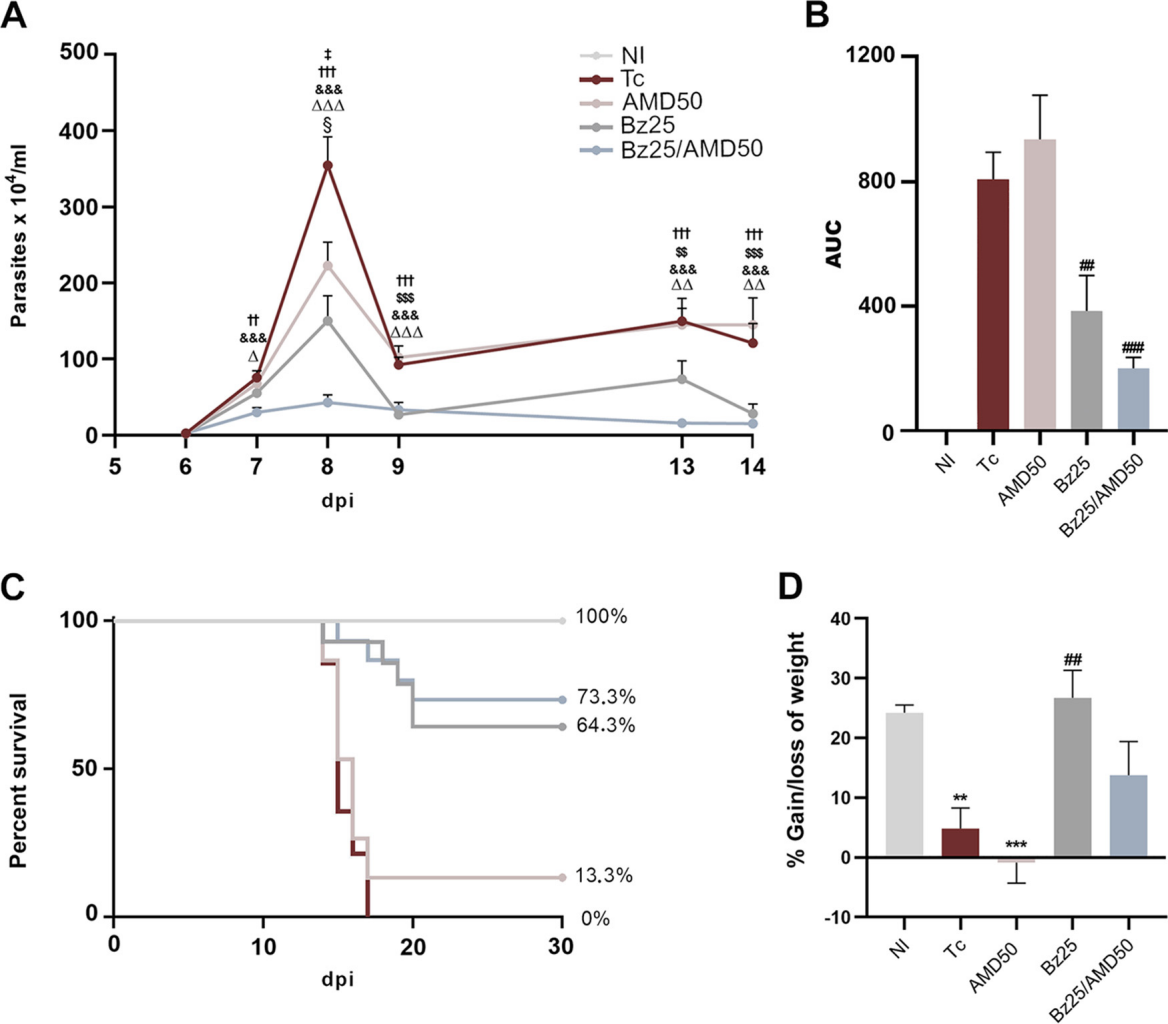
Administration of Bz/AMD reduces parasite load, increases survival of T. cruzi-infected mice, and does not alter body weight. (A) Parasitemia curve; (B) area under the curve (AUC) of parasitemia (5 to 14 dpi); (C) percentage of survival; (D) percentage of body weight gain/loss at 14 dpi in relation to day 5. #, different from Tc; *, different from NI; §, Tc versus AMD50; Δ, Tc versus Bz25; &, Tc versus Bz25/AMD50; $, AMD50 versus Bz25; †, AMD50 versus Bz25/AMD50; ‡, Bz25 versus Bz25/AMD50. # ‡ § Δ, *P* < 0.05; ** ## †† ΔΔ $$, *P* < 0.01; *** ††† ΔΔΔ $$$ &&&, *P* < 0.001.

First, we examined the effect of the therapeutic regimens on the parasitemia curve (Fig. 1A). We observed a significant decrease in the parasitemia peak in the three treated groups compared to positive-control infected mice (Tc) (Fig. 1A), while in area under the curve (AUC) analysis only the Bz25 group (receiving 25 mg/kg body weight/day Bz25) and the Bz25/AMD50 (50 mg/kg/day AMD) group were decreased in comparison to Tc (Bz25/AMD25, 199.2 and Bz25, 383.4 versus Tc, 806.4; AUC of parasitemia, *P* < 0.01) (Fig. 1B). The Bz25/AMD50 and Bz25 groups presented a consistent decrease in parasitemia compared to the Tc (Fig. 1A). The parasitemia peak of the combination group showed a 3-fold decrease compared to monotherapy with Bz (Bz25/AMD50, 43.02 versus Bz25, 150.20; parasites × 10^4^/mL, *P* = 0.021) (Fig. 1A). In initial experiments conducted with a lower AMD dose (25 mg/kg/day), the Bz25 and Bz25/AMD25 groups showed similar efficacy in reducing the parasitemia peak compared to the Tc group (Bz25/AMD25, 166.9 and Bz25, 150.20 versus Tc, 354.5; parasites × 10^4^/mL, *P* < 0.01), while AMD25 did not decrease parasitemia (AMD25, 315.9 versus Tc, 354.5; parasites × 10^4^/mL, *P* > 0.99) (Fig. S1). Therefore, the analysis of both AMD25 and Bz25/AMD25 was discontinued.

The survival curves show that in the Tc group, mouse death started at 16 days postinfection (dpi), and all animals died at 18 dpi. The percentages of survival at 30 dpi were 13.3%, 64.3%, and 73.3% for AMD50, Bz25 and Bz25/AMD50, respectively (Fig. 1C). At 14 dpi, the Tc and AMD50 groups showed decreased body weight (BW) gain compared to uninfected mice (negative-control group; NI) (Fig. 1D), and this decrease was prevented by treatment with Bz alone or combined with AMD (Fig. 1D; NI, 24.21%; Tc, 4.87%; AMD50, 20.86%; Bz25, 26.75%; Bz25/AMD50, 13.79%; change in body weight).

### Combination Bz/AMD prevents heart damage in T. cruzi-infected mice.

**FIG 2:**
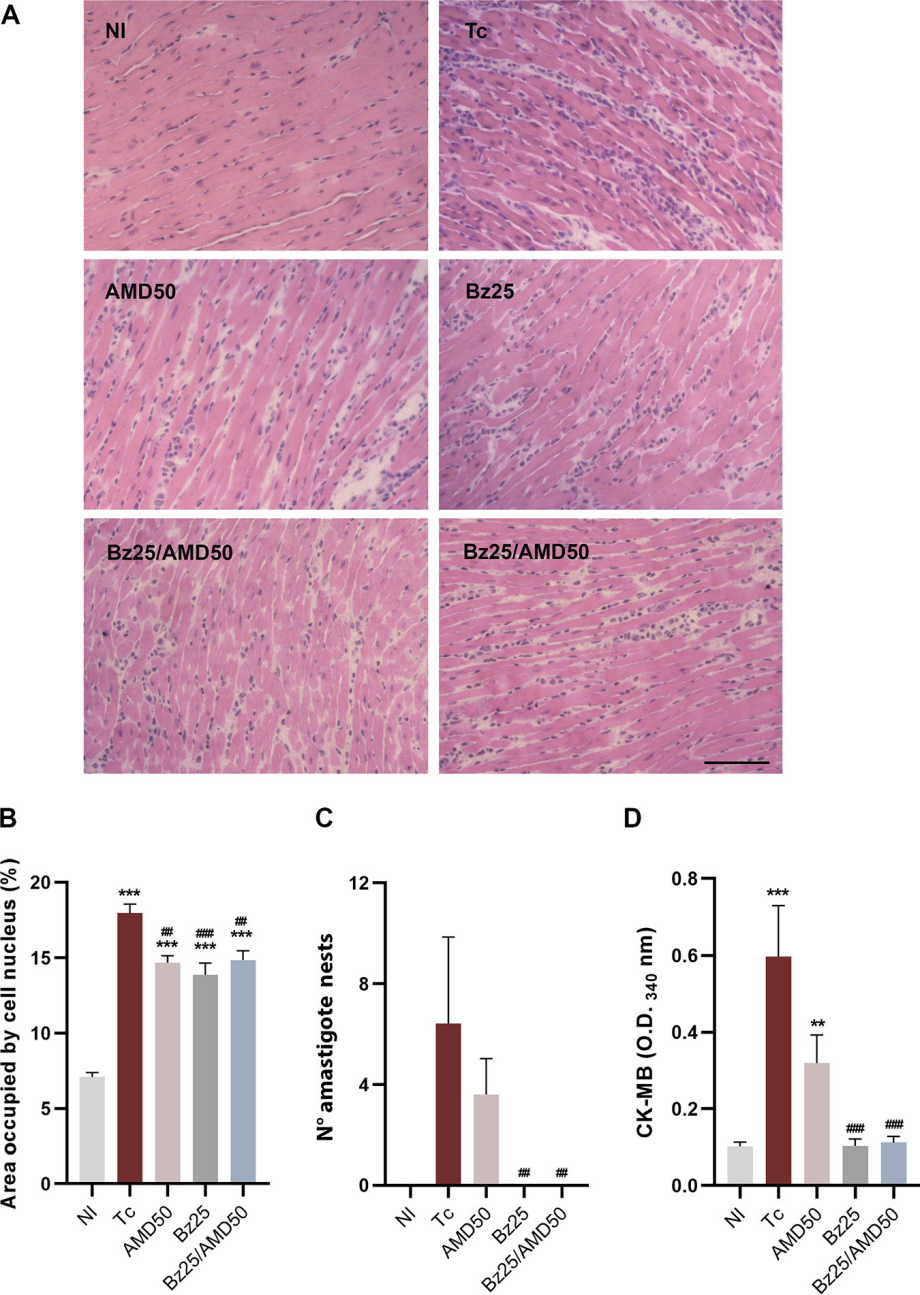
Combined Bz/AMD treatment prevents cardiac damage in T. cruzi-infected mice. (A) Representative photomicrograph of cardiac tissue from T. cruzi-infected mice showing an increase in inflammatory foci and loss of cardiac fiber integrity. (B) Percentage area occupied by cell nuclei. (C) Number of parasite nests in cardiac tissue. (D) Activity of plasmatic CK-MB. *, different from NI; #, different from Tc. ** ##, *P* < 0.01; *** ###, *P* < 0.001. Bar = 500 μm.

Myocardial sections from the Tc group had numerous diffusely distributed foci of inflammation, often associated with necrotic areas, amastigote nests, and loss of cardiac fiber integrity (Fig. 2A to C). Treatment with Bz, AMD, and Bz/AMD significantly reduced the number of cell nuclei stained (hematoxylin) compared to Tc, indicating reduced inflammation in cardiac tissue (Fig. 2B; Tc, 17.97% of area occupied by cell nuclei versus AMD50, 14.65%; Bz25, 13.87%; Bz25/AMD50, 14.84%; *P* < 0.01). Moreover, compared to Tc, a significant reduction in the number of amastigote nests was observed in the Bz25 and Bz25/AMD50 groups (Fig. 2C; Tc, 6.42 amastigote nests versus Bz25, 0.0 and Bz25/AMD50, 0.0; *P* < 0.001). Additionally, in comparison to Tc, the activity of creatine kinase isotype MB (CK-MB) in plasma was significantly lower in Bz25 and Bz25/AMD50 (Fig. 2D; Tc, 0.59 optical density at 340 nm versus Bz25, 0.10 and Bz25/AMD50, 0.01; *P* < 0.0001).

**FIG 3:**
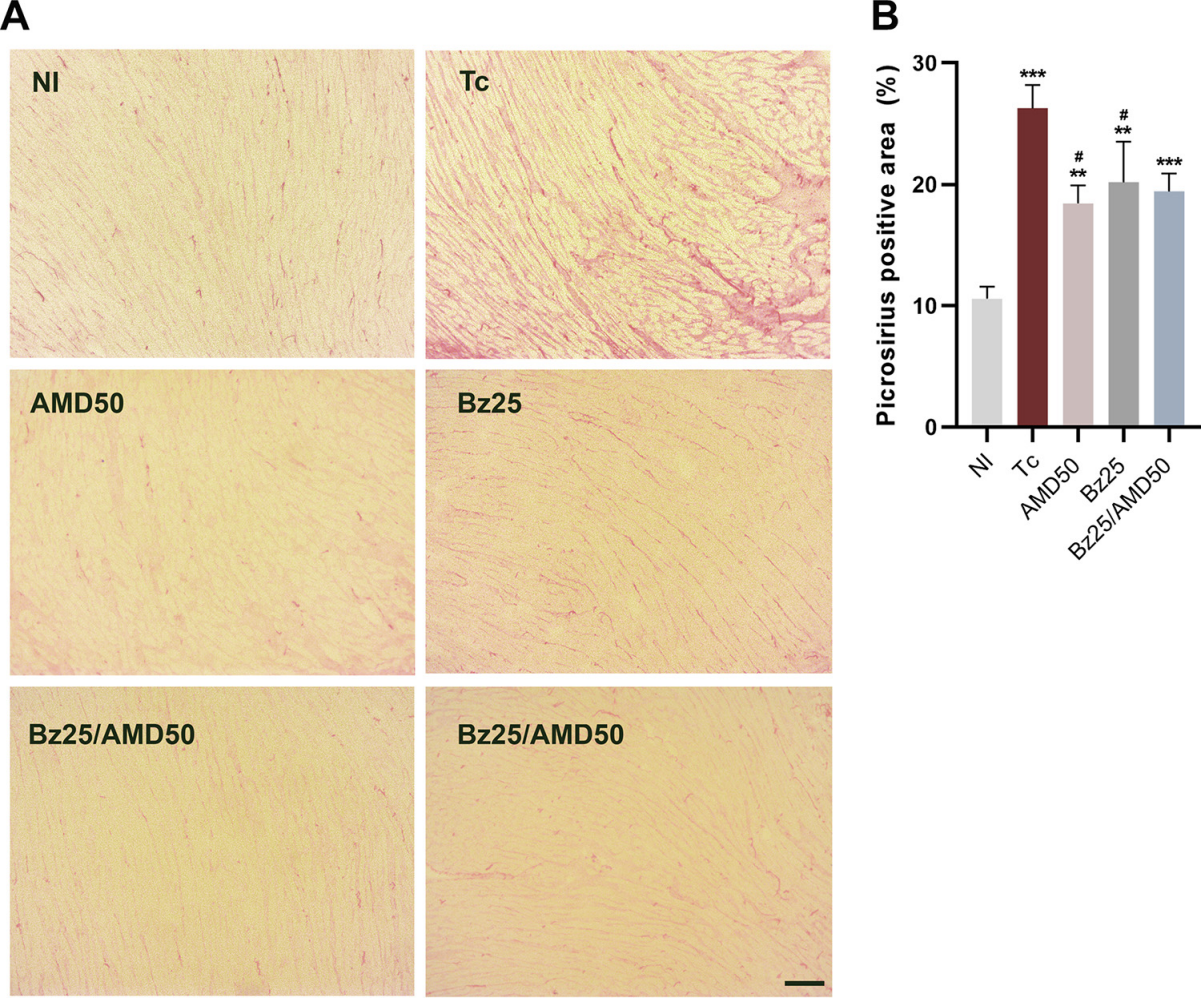
Combined Bz/AMD schemes ameliorate fibrosis in cardiac tissue. (A) Representative photomicrographs of cardiac tissue of T. cruzi-infected mice showing an increase in collagen deposition. (B) Quantification of the area positive for collagen. *, different from NI; #, different from Tc. #, *P* < 0.05; ** ##, *P* < 0.01; ***, *P* < 0.001. Bar = 500 μm.

Acutely, T. cruzi-infected mice (Tc) showed increased collagen deposition in cardiac tissue, as assessed by picrosirius staining, compared to NI (Fig. 3A and B). Compared to Tc, collagen deposition was significantly reduced in the AMD50 and Bz25 groups (Fig. 3B; Tc, 26.27 % of area positive for collagen, versus AMD50, 18.45% and Bz25, 20.17%; *P* < 0.05).

### Bz/AMD administration restores electrical abnormalities in T. cruzi-infected mice.

**FIG 4:**
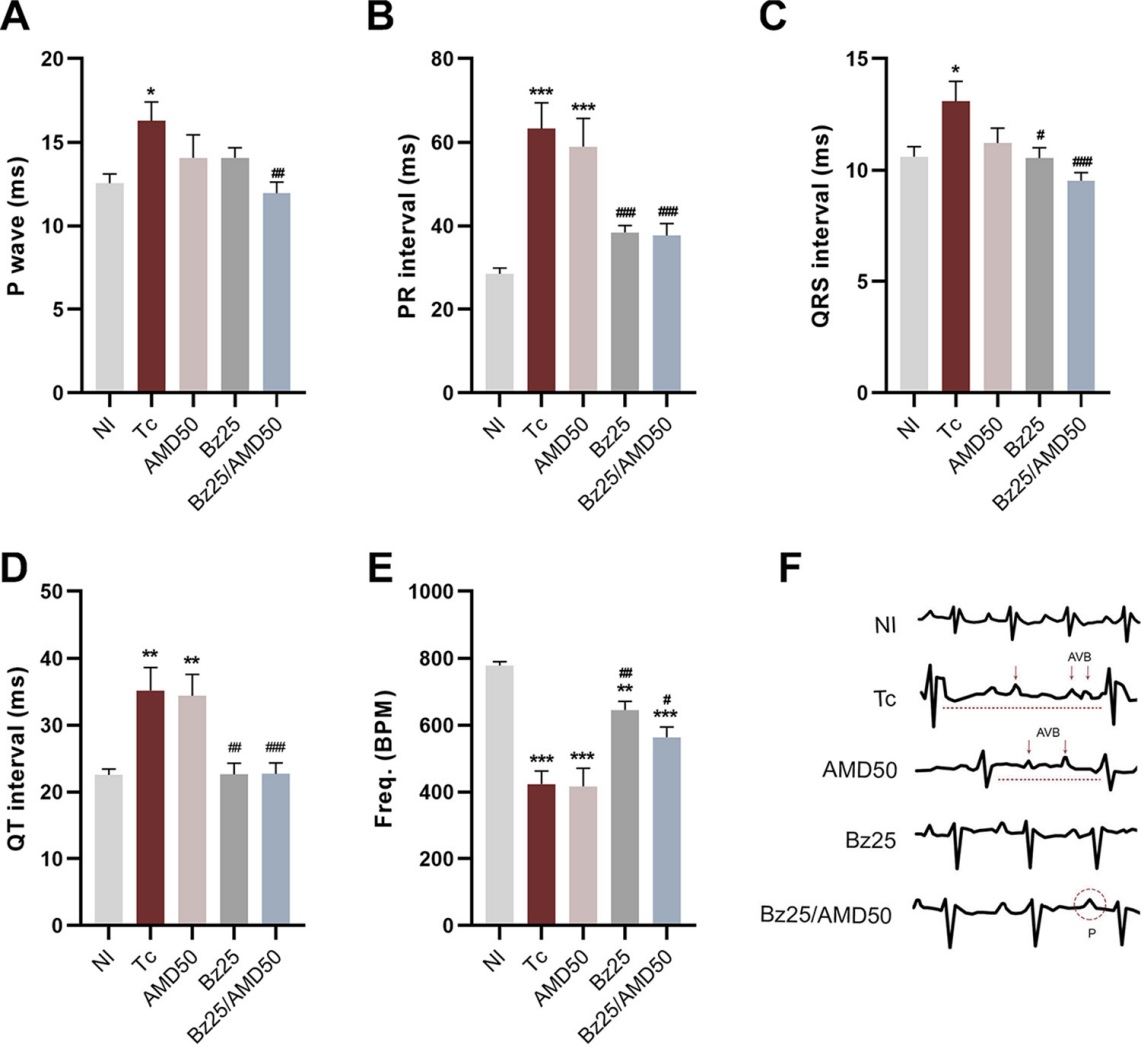
Combined Bz/AMD administration reduces electrical abnormalities. (A to E) Electrocardiography (ECG) of T. cruzi-infected mice at 14 dpi showing (A) P wave duration, (B) PR interval, (C) QRS interval, (D) QT interval in milliseconds (ms), and (E) cardiac frequency in heartbeats per minute (BPM). (F) Representative ECG tracings. #, different from Tc; *, different from NI. * # $, *P* < 0.05; ** ##, *P* < 0.01; *** ###, *P* < 0.001.

The combination of Bz/AMD significantly reduced the duration of the P wave compared to Tc (Tc, 16.29 P wave duration in ms versus Bz25/AMD50, 11.94 P wave duration in ms; *P* = 0.006) (Fig. 4A). Treatment with Bz alone or in combination with AMD significantly reduced the PR (extends from the beginning of the P wave until the beginning of the QRS complex), QRS (a combination of the Q wave, R wave and S wave), and QT intervals (Fig. 4B to D) and prevented bradycardia compared to Tc (Fig. 4E). AMD monotherapy prevented the increase in P wave duration and QRS interval associated with T. cruzi infection (Fig. 4A and C).

Combined treatment with Bz25 and Bz25/AMD50 was most effective in preventing pathological changes in the cardiac electrical conduction system, as evidenced by the lowest percentages of sinus arrhythmia (ART) (6.25 and 5.5%), atrioventricular block (AVB) (18.75 and 16.6%), and bradycardia (BRA) (25 and 22.2%) (Table 1).

**TABLE 1:** Combination therapy with Bz/AMD ameliorates electrical abnormalities in acute T. cruzi-infected mice

Type of arrhythmia^*a*^	Data for treatment groups:^*b*^				
	NI	Tc	AMD50	Bz25	Bz25/AMD50
ART (%)	0 ##	37.5	38.5	6.25 #	5.55 #
BRA (%)	0 ###	56.3	92.31#	18.75 #	16.66 #
AVB (%)	0 ####	75.0	69.2	25.0 ##	22.2 ##

a Percentage of animals with cardiac arrhythmia events. ART, sinus arrhythmia; AVB, atrioventricular block; BRA, bradycardia.

b Different from Tc; #, *P* < 0.05; ##, *P* < 0.01; ###, *P* < 0.001; ####, *P* < 0.0001.

### Bz/AMD reduces inflammatory cytokines in the hearts of T. cruzi-infected mice.

**FIG 5:**
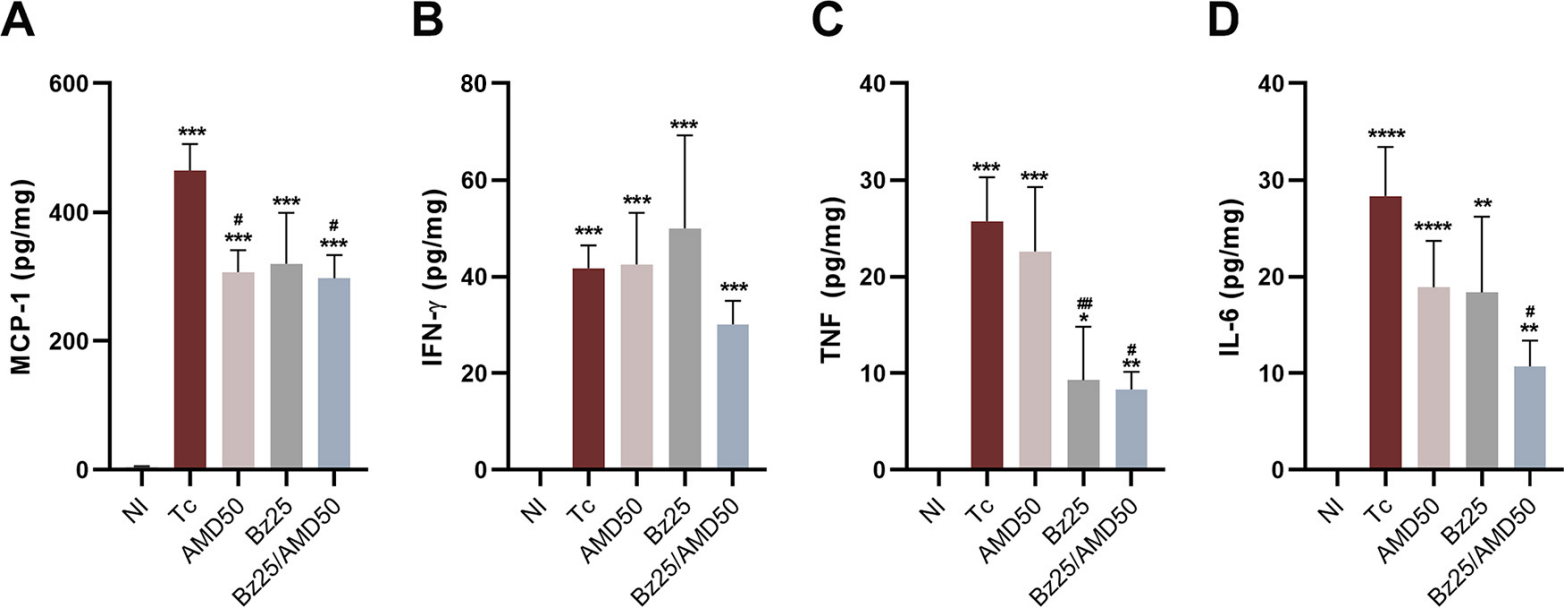
Combined Bz/AMD administration reduces inflammatory cytokines in cardiac tissue. Cytokine production of the T. cruzi-infected mice at 14 dpi. (A) MCP-1; (B) IFN-γ; (C) TNF, and (D) IL-6. Data are expressed in pg cytokine/mg of total protein. *, different from NI; #, different from Tc. * #, *P* < 0.05; ** ##, *P* < 0.01; ***, *P* < 0.001; ****, *P* < 0.0001.

T. cruzi-infected mice showed increased MCP-1, interferon-γ (IFN-γ), tumor necrosis factor (TNF) and interleukin-6 (IL-6) levels compared to NI mice (Fig. 5A to D). The AMD50 and Bz25/AMD50 groups presented decreased MCP-1 levels in relation to Tc (Fig. 5A; Tc, 465.0 pg/mg of total protein versus AMD50, 307.2 and Bz25/AMD50, 297.3; *P* < 0.05). None of the treatments influenced cardiac IFN-γ cytokine levels (Fig. 5B). Furthermore, in groups Bz25 and Bz25/AMD50, the treatment partially protected the heart from the increase in TNF concentration induced by T. cruzi infection (Fig. 5C; Tc, 41.76 pg/mg of total protein versus Bz25, 50.02 and Bz25/AMD50, 30.17; pg/mg; *P* < 0.05). Finally, compared to the Tc group, the IL-6 concentration was decreased in the Bz25/AMD50 group (Tc, 28.31 pg/mg of total protein versus Bz25/AMD50, 10.72; *P* < 0.05) (Fig. 5D).

### Combined Bz/AMD treatment restores Cx43 immunoreactivity.

**FIG 6:**
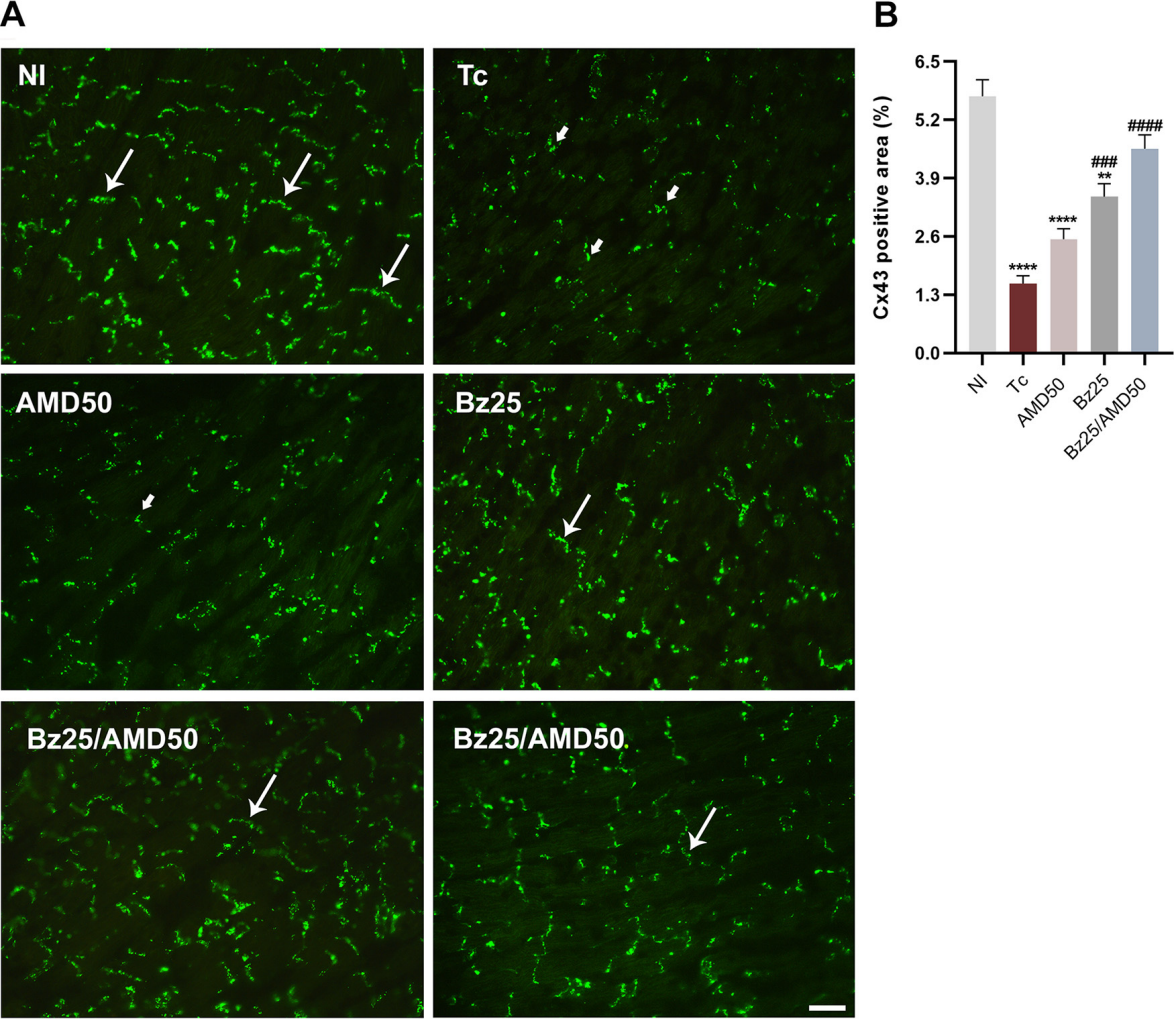
Combined Bz/AMD administration ameliorates gap junction integrity in cardiac tissue. (A) Representative photomicrographs of cardiac tissue sections of T. cruzi-infected mice stained for Cx43 (in green). Thin white arrows indicate regular gap junction plaques, and thick small arrows indicate disrupted plaques. (B) Quantification of the percentage of Cx43-positive area in heart tissue. *, different from NI; #, different from Tc. **, *P* < 0.01; ###, *P* < 0.001; **** ####, *P* < 0.0001. Bar = 10 μm.

We observed that infected mice had a decrease in the Cx43 immunoreactivity in the intercalated disks of cardiac tissue compared to NI (Fig. 6A and B). AMD did not restore Cx43 expression in cardiac tissue (Fig. 6; Tc, 1.55% of Cx43-positive area versus AMD50, 2.54%; *P* > 0.05). Compared to the Tc group, the Bz25 group had increased Cx43 expression (Tc, 1.55% of Cx43-positive area versus Bz25, 3.49%; *P* = 0.0001). However, only Bz25/AMD had a similar Cx43 signal compared to NI in the cardiac tissue (NI, 5.72% of Cx43-positive area versus Bz25/AMD50, 4.56%; *P* > 0.05).

### Bz/AMD reduces liver damage caused by acute experimental T. cruzi infection.

**FIG 7:**
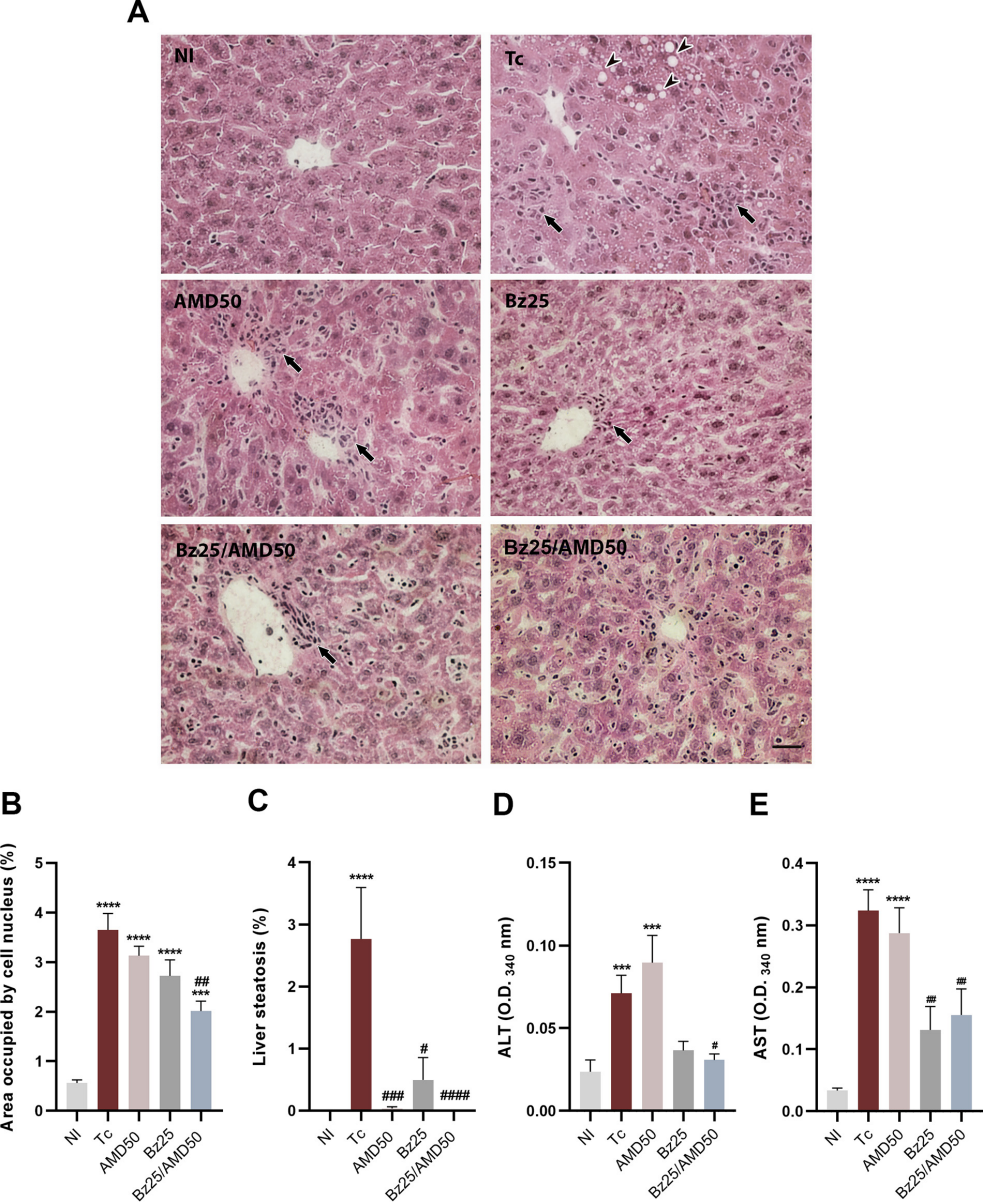
Combined Bz/AMD reduces liver damage caused by acute experimental T. cruzi infection. (A) Representative photomicrography of liver tissue of T. cruzi-infected Swiss mice showing an increase in inflammatory cells (black arrows) and ectopic fat deposits (arrowheads). (B) Percentage area occupied by cell nuclei. (C) Percentage area positive for ectopic fat deposits. (D and E) Activity of (D) ALT and (E) AST assessed in the plasma. #, different from Tc; *, different from NI. #, *P* < 0.05; ##, *P* < 0.01; *** ###, *P* < 0.001; **** ####, *P* < 0.0001. Bar = 20 μm.

Compared to NI animals, T. cruzi-infected animals (Tc) showed morphological changes characteristic of nonalcoholic steatohepatitis (NASH) (Fig. 7A). Histopathological analysis showed high numbers of inflammatory cells concentrated in the periportal spaces and sinusoidal dilatation in untreated and treated animals. Compared to Tc, only the combination Bz/AMD significantly reduced the number of cell nuclei stained (hematoxylin), indicating reduced inflammation in hepatic tissue (Tc, 3.65% of area occupied by cell nuclei versus Bz25/AMD50, 2.01%; *P* = 0.009) (Fig. 7B). In addition, treatment with Bz, AMD, and Bz/AMD significantly reduced ectopic fat deposits, indicating that all treatment regimens improved steatosis (Fig. 7C; Tc, 2.76% of area occupied by cell nucleus versus AMD50, 0.03%; Bz25, 0.49; Bz25/AMD50, 0%; *P* < 0.05). Compared to infected untreated mice, Bz25/AMD50 significantly reduced the activity of ALT (Tc optical density at 340 nm [OD_340_], 0.07 versus Bz25/AMD50 OD_340_, 0.03; *P* < 0.05) and AST (Tc OD_340_, 0.323 versus Bz25/AMD50 OD_340_, 0.155; *P* = 0.009), while Bz25 only reduced AST activity (Tc OD_340_, 0.323 versus Bz25 OD_340_, 0.13; *P* = 0.044) (Fig. 7D and E).

## DISCUSSION

AMD was originally commercialized as a coronary dilatator for the treatment of angina pectoris (21, 22). However, its use as a type III antiarrhythmic drug (in agreement with the Vaughan Williams classification) was later recognized by the U.S. Food and Drug Administration (FDA) in 1985 (23). Currently, AMD is the most commonly used drug to treat patients with CD-associated cardiac arrhythmias (13, 24). The repurposing of this drug was first thought to be an antifungal drug against Cryptococcus, Aspergillus, and *Candida* spp. (25). Subsequently, the efficacy of AMD and its derivatives against different pathogenic trypanosomatids, including T. cruzi, Trypanosoma brucei, and *Leishmania* spp. was described (26–28).

In the present work, we observed that T. cruzi-infected mice treated with AMD showed a reduction in parasitemia, which is in agreement with Benaim et al. (26), who described for the first time the *in vivo* trypanocidal effect of AMD. However, the most effective therapeutic regimen in reducing parasitemia and increasing mouse survival was observed in the Bz25/AMD50 group. These results indicate a possible pharmacological interaction between Bz and AMD, which could be due to different mechanisms of action and may favor the elimination of the parasite and improve the prognosis of the disease (6). The mechanism of action of AMD against T. cruzi is related to the inhibition of ergosterol biosynthesis (26). In addition, AMD has been shown to cause disruption of intracellular Ca^2+^ homeostasis in T. cruzi (26, 28). The mechanism of action of Bz has not been fully elucidated, but it is known that it is degraded anaerobically to form an intermediate metabolite that covalently binds parasite macromolecules such as lipids, DNA, and proteins (29, 30).

The reduction in parasitemia observed in the AMD50, Bz25, and Bz25/AMD50 treatment groups may also be related to lower collagen deposition in cardiac tissue. Cardiac injury in the acute phase of CD involves intense tissue parasitism that causes myocyte destruction by inflammatory cells, leading to reparative fibrosis, which is a remarkable feature of CC in humans and mouse models (31, 32). Consistently, all treatment regimens reduced excessive collagen deposition, inflammation in cardiac tissue, and the activity of CK-MB, a plasmatic biomarker of myocardial injury.

Electrical abnormalities were also observed during acute T. cruzi infection in mice. At 14 dpi, the ECG of infected animals showed significantly higher P-wave, PR, QRS, and QT intervals than uninfected mice. Relevant electrical abnormalities found in patients with CC, such as atrioventricular block (AVB) and bradycardia (BRA), were also reproduced in the mouse model presently used (33). Interestingly, we showed that in AMD50, the treatment prevented the increase in P wave and QRS interval observed in T. cruzi-infected animals and therefore can be considered a good marker of cardiac function improvement, as prolonged QRS intervals have been associated with fibrosis severity and reduction in left ventricular ejection fraction in CD patients (34–36). On the other hand, the AMD50 group induced a higher percentage of mice with bradycardia than the Tc group, which could be a side effect of this antiarrhythmic drug, as previously described in clinical studies (23). Interestingly, this side effect seems to have been abolished by its combination with 25 mg/kg Bz, as the Bz25/AMD50 group had the lowest percentage of bradycardia.

The Bz25/AMD50 group was the most effective therapeutic regimen in preventing electrical abnormalities caused by acute infection. This group was the only group to significantly reduce the duration of the P-wave, indicating an improvement in atrial function of the infected animals. It has also been reported that abnormal P waves are associated with increased all-cause mortality in patients with CD (35). Moreover, Bz25/AMD50 showed the lowest incidence of arrhythmia, including AVB and BRA.

The anti-inflammatory effect of AMD has already been reported (36–38); however, the present study is the first to describe the anti-inflammatory effect of AMD in the acute phase of CD, as evidenced by the reduction in the concentration of the chemokine MCP-1 and of inflammatory infiltrates in the cardiac tissue of infected mice. These results could be related to the trypanocidal activity of AMD, as there was a significant reduction in the parasitemia peak of AMD50 animals compared to Tc.

Treatment with the Bz/AMD combination also decreased the levels of IFN-γ and TNF in cardiac tissue, whereas Bz monotherapy only decreased TNF compared with Tc. IFN-γ and TNF cytokines are essential for the control of parasitemia and for the survival of infected animals in the acute phase (39, 40). However, the high production of IFN-γ and TNF in the acute phase has also been associated with the development of CCC (41–45). Therefore, although these cytokines favor parasite elimination, their production at high levels is thought to lead to the maintenance of tissue damage caused by the proinflammatory stimulus and to the increased production of reactive oxygen species (ROS), which contributes to CCC progression (46–48). In this context, our results suggest that the reduction in IFN-γ and TNF observed in Bz25/AMD50 was beneficial, as there was a decrease in cardiac tissue inflammation without affecting the trypanocidal efficacy of the treatment.

We also showed that only in the Bz25/AMD50 group did a significant decrease in the levels of IL-6 in cardiac tissue occur compared with Tc. This result can be considered an important therapeutic advantage of the combination, as the endothelial damage caused by T. cruzi infection is directly related to the increased production of IL-6 (5). The functional alteration of endothelial cells caused by infection is considered to be one of the main factors contributing to the deterioration of microcirculation and reduction of tissue perfusion in CD (49–51). An increase in IL-6 concentration has already been observed in patients developing the severe form of CCC and has also been associated with an increased risk of sudden death (43, 52). In addition, Lazzerini et al. described that in patients with acute myocarditis, the increase in IL-6 promotes atrial remodeling, with a decrease in Cx40 and Cx43 expression, leading to dysfunction of gap junctions in cardiac tissue. A relationship between the increase in IL-6 levels and electrocardiographic changes in the P wave and atrial fibrillation has also been previously described (53).

It is well documented that T. cruzi infection is associated with decreased connexin-43 expression in cardiac tissue (19, 54). Accordingly, our results suggest that the significant shortening of the duration of the P wave in the Bz25/AMD50 group may be associated with a decrease in the concentration of IL-6 and with an increase in the expression of connexin-43 in cardiac tissue. The integrity of gap junctions observed in Bz25/AMD50 could represent an important clinical advantage because electrical activation of the myocardium depends on the integrity of these cellular connections (55). In addition, alterations in the density and function of gap junctional communication are currently thought to contribute to the electrophysiological disturbances observed in patients with CCC (16, 51).

The impairment of liver function is a hallmark of T. cruzi acute infection (20, 56). Moreover, because Bz and AMD are metabolized in the liver, we investigated whether their concomitant use could exacerbate the liver injury caused by the infection (57, 58). However, no hepatotoxic effect was observed in Bz25/AMD50-treated mice, as shown by the reduction in morphological abnormalities and decreased steatosis and transaminase levels compared to those of untreated and T. cruzi-infected mice.

To our knowledge, this is the first paper to evaluate the effect of Bz and AMD combined therapy for CD treatment. We conclude that coadministration of Bz and AMD could be considered an alternative strategy for the treatment of symptomatic cardiomyopathy, as the combined drugs improved parasitism, mouse survival, and cardiac function, likely due to the reduction in systemic immune activation, electrical abnormality restoration, and electrical abnormality and cardiac conexin-43 expression restoration. In addition, combined therapy allowed Bz to be used at lower doses, which may attenuate the toxic effects associated with this drug (59). Further studies are under way to investigate the effect of the Bz/AMD combination in experimental CCC.

## MATERIALS AND METHODS

### Drugs.

Benznidazole (Bz) was purchased from Laboratório Farmacêutico do Estado de Pernambuco (Recife, Pernambuco, Brazil), and amiodarone hydrochloride (AMD) was purchased from Sanofi-Aventis (Paris, France). For the *in vivo* tests, solutions of Bz and AMD were prepared daily in distilled and sterile water as previously described (60).

### *In vivo* assays.

Mice (5-week-old male Swiss Webster outbred mice) were obtained from the animal facility of the Instituto de Ciência e Tecnologia em Biomodelos (ICTB, Rio de Janeiro, Brazil). Five mice were housed per cage and were kept in a conventional room at 20 to 24°C under a 12 h/12 h light/dark cycle. The animals were provided sterilized water and chow *ad libitum*. Three independent experiments were performed. After individual analysis and finding the reproducibility of the data, the results were grouped. All procedures were carried out in conformity with the guidelines established by the FIOCRUZ Committee of Ethics for the Use of Animals (license L-038/2018).

### Mouse infection and treatment schemes.

**FIG 8:**
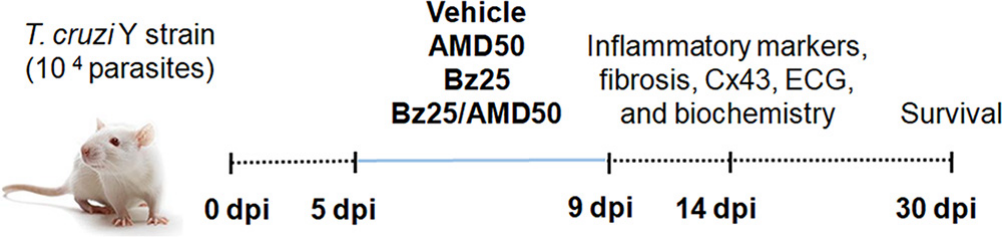
Acutely T. cruzi-infected Swiss male mice were treated by gavage daily (5 to 9 days postinfection [dpi]) with vehicle (NI and Tc), AMD (50 mg/kg [AMD50]), Bz (25 mg/kg [Bz25]), or Bz plus AMD (Bz25/AMD50). Some of the animals were euthanized and analyzed at 14 dpi (*n* = 5 to 15 per group), and 5 animals/group were monitored up to 30 dpi for survival analysis (*n* = 15). This treatment scheme was used in three independent experiments. ECG, electrocardiography; Cx43, connexin-43; Bz, benznidazole; AMD, amiodarone.

Male mice (*n* = 5 to 15 per group, three independent assays) were inoculated intraperitoneally (i.p.) with 10^4^ T. cruzi bloodstream trypomastigotes (BT) of the Y strain (DTU II) and were treated by gavage in schemes of mono (Bz or AMD) and combined (BZ/AMD) therapy. Consistent with previous studies (61, 62), we performed a short course of treatment for 5 consecutive days (once a day), starting on the 5th day postinfection (dpi), corresponding to the onset of parasitemia in this experimental acute model (33). The monotherapy groups were Bz25, receiving 25 mg/kg/day Bz, and AMD50, receiving 50 mg/kg/day AMD. The combined therapy group, Bz25/AMD50, received 25 mg Bz/kg/day plus 50 mg AMD/kg/day. The dose of 25 Bz mg/kg is well established as a suboptimal dose (60), which corresponds to a one-quarter Bz optimal dose (100 mg Bz/kg/day) (63). A dose of 50 mg/kg/day of AMD was chosen based on the estimated body surface area of mice and the average body surface area of an adult human and is considered equivalent to the clinical dosage for an adult human (300 mg/kg/day) (64). Additionally, half of the dose of AMD (AMD25 [25 mg/kg/day]) and its combination with the suboptimal dose of Bz (Bz25/AMD25 [Bz 25 mg/kg/day and AMD 25 mg/kg/day]) were tested. The two control groups were NI (uninfected mice, negative control) and Tc (infected mice, positive control), both treated with vehicle (water). Only animals with positive parasitemia were included in the study. The analyses were performed at 14 dpi because electrical abnormalities and heart injury are more prominent at this point (33). Some of the animals in each experimental group were followed up to 30 dpi for survival analysis. The experimental design is depicted in Fig. 8.

### Parasitemia, body weight, and mortality.

Parasitemia was individually checked by direct microscopy using the Pizzi-Brener method (65). Briefly, 5 μL of blood was collected and mounted on a slide with a coverslip, and 50 fields were randomly counted. Body weight was evaluated at the beginning of treatment (5 dpi) and at 14 dpi using a precision balance. The result was expressed as percentage weight gain/loss, which corresponds to the change in weight from the start of treatment to the endpoint of the experiment. The cumulative mortality was registered daily, and the survival rate was calculated at 30 dpi.

### Biochemical analysis.

Blood was collected at 14 dpi by cardiac puncture after euthanasia of the animals for plasma collection. Liver injury was assessed using alanine aminotransferase (ALT) and aspartate aminotransferase (AST), while cardiac injury was quantified by creatine kinase isotype MB (CK-MB). Commercial kits were used, and the manufacturer’s recommendations were followed (LabTest Laboratory, Minas Gerais, Brazil).

### ECG analysis.

ECG recordings and analysis were performed in physically restrained (nonsedated) mice at 14 dpi, according to previous studies (66). Briefly, all mice were fixed in the supine position, and transducers were carefully placed under the skin in accordance with the preferential derivation (DII). The ECG parameters analyzed were variation at the P wave and PR, QRS and QT intervals measured in ms, and heart rate monitored by beats/min (bpm).

### Histopathological analysis.

At 14 dpi, heart and liver tissues were collected for histopathological analysis. Tissues were embedded in Tissue-Tek OCT (Sakura, Torrance, USA), frozen in liquid nitrogen and stored at −80°C. Then, 3-μm-thick cardiac sections fixed in a 4% paraformaldehyde solution for 30 min at room temperature were obtained using a cryostat (CM1850; Leica Biosystems, Wetzlar, Germany). For histopathological analysis, the slices were stained with hematoxylin and eosin (H&E) or picrosirius red. At least 5 fields/sample were evaluated by light microscopy (AxioLab A1; Zeiss, Oberkochen, Germany). The percentages of area (i) positive for collagen, (ii) occupied by cell nucleus, and (iii) positive for steatosis were quantified using Image J (NIH, EUA) and STEPanizer (Oracle Corporation, Redwood Shores, CA, USA), as previously described (67, 68).

### Immunofluorescence.

For connexin-43 (Cx43) expression evaluation, cardiac sections (3 μm thick) were fixed in a 4% paraformaldehyde solution for 30 min at room temperature. After the sections were washed in PBS and permeabilized with 0.5% Triton X-100, nonspecific staining was blocked with bovine serum albumin (BSA). Incubation with the primary antibody (diluted 1:2,000; Sigma-Aldrich, St. Louis, USA) was performed for 1 h at 37°C in blocking buffer (PBS supplemented 2% BSA). Then, all samples were incubated for 1 h with the secondary antibody (1:2,000, anti-rabbit Alexa Fluor 488; Sigma-Aldrich). At least 5 fields/sample were evaluated by fluorescence microscopy (AxioLab A1; Zeiss). The percentage of the area positive for Cx43 was evaluated using Image J (NIH).

### Cardiac cytokine analysis.

Cardiac tissue was collected at 14 dpi and kept frozen at −80°C until analysis. Cardiac fragments were thawed and disrupted by sonication in ice-cold extraction buffer (PBS supplemented with 1% protease cocktail inhibitor) (Roche, Basel, Switzerland) and 1% Nonidet P40 (Sigma-Aldrich). Afterward, all samples were centrifuged at 500 × *g*, and the supernatants were collected and used for cytokine analysis (69). Monocyte chemoattractant protein-1 (MCP-1), interferon-γ (IFN-γ), tumor necrosis factor (TNF), and interleukin-6 (IL-6) were measured using the BD cytometric bead array (CBA) mouse inflammation kit (Becton, Dickinson, New Jersey, USA) according to the manufacturer’s recommendations using a FACSCalibur device (Becton, Dickinson). Data analysis was performed using FCAP software (Becton, Dickinson). Protein concentration was determined using the Pierce BCA protein assay kit (Thermo Fisher, Waltham, MA, USA).

### Statistical analysis.

Results were expressed as means ± standard error of the mean (SEM) for each group. The normality of the distribution of the variables was tested with Shapiro-Wilk tests and confirmed with Q-Q plots. Between-group comparisons were made using one-way analysis of variance (ANOVA) followed by Tukey’s *post hoc* test, Kruskal-Wallis test followed by Dunn’s *post hoc* test, and/or qui-square test (InStat 8.0; GraphPad Software, Inc., La Jolla, CA, USA). *P* values of <0.05 were considered significant.
