# Supplementary material for: Experimental Combination Therapy with Amiodarone and Low-Dose Benznidazole in a Mouse Model of Trypanosoma cruzi Acute Infection
Source: Microbiol Spectr. 2022 Feb 9;10(1):e01852-21. doi: 10.1128/spectrum.01852-21 (PMC8826820; doi:10.1128/spectrum.01852-21)
Supplement: SUPPLEMENTAL FILE 1 — Supplemental material. Download SPECTRUM01852-21_Supp_1_seq1.pdf, PDF file, 0.2 MB [file spectrum01852-21_supp_1_seq1.pdf]

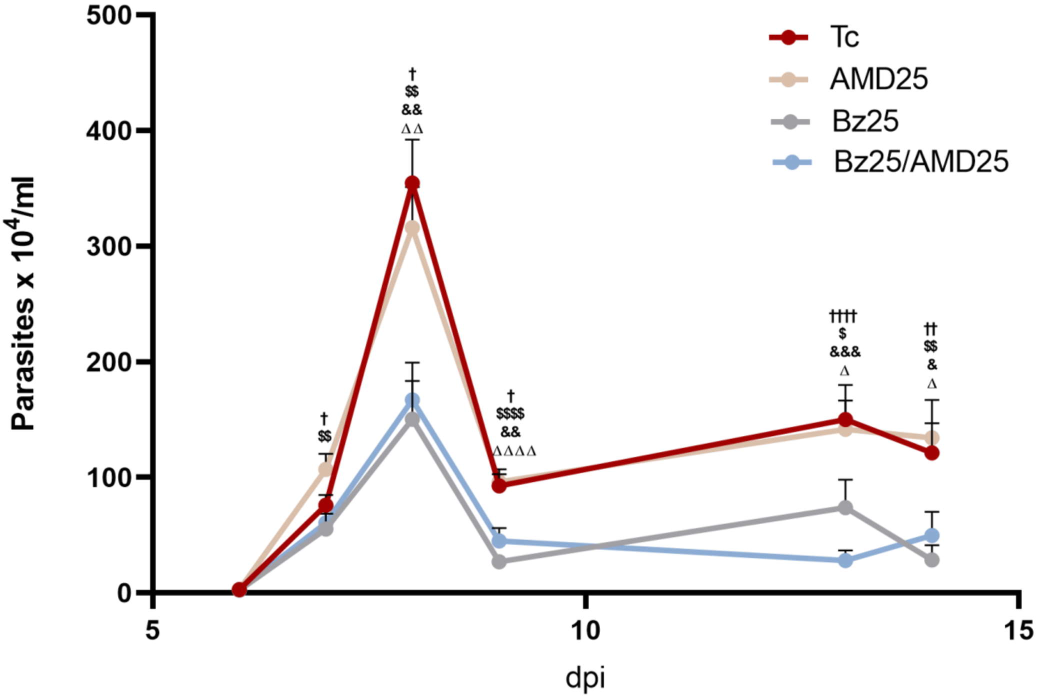

Supplementary Fig. S1: Parasitemia curve of each experimental group. Error bars indicate mean±SEM. Δ: **Tc** *vs.* **Bz25**; &: **Tc** *vs.* **Bz25/AMD25**; $: **AMD25***vs.* **Bz25**; †: **AMD25***vs.* **Bz25/AMD25**; p range: † Δ $ &: p<0.05; †† ΔΔ $$&&: p<0.01; &&&: p<0.001; ††††ΔΔΔ $$$$: p <0.0001.
